# Ultrasensitive Ti_3_C_2_Tx@Pt-Based Immunochromatography with Catalytic Amplification and a Dual Signal for the Detection of Chloramphenicol in Animal-Derived Foods

**DOI:** 10.3390/foods13091416

**Published:** 2024-05-05

**Authors:** Mengfang Lin, Zhimin Gao, Zhenjie Qian, Youwen Deng, Yanhong Chen, Yu Wang, Xiangmei Li

**Affiliations:** 1 Guangdong Provincial Key Laboratory of Food Quality and Safety, College of Food Science, South China Agricultural University, Guangzhou 510642, China; 15978008477@163.com (M.L.); d635921341@163.com (Y.D.); 2 Guangdong Agricultural Product Quality and Safety Center (Guangdong Green Food Development Center), Guangzhou 510230, China; 18922147530@163.com; 3 Guangzhou Institute for Food Inspection, Guangzhou 511410, China; qianzhenjie@hotmail.com (Z.Q.); chenyanhom@outlook.com (Y.C.)

**Keywords:** Ti_3_C_2_Tx@Pt, catalytic amplification, chloramphenicol, immunochromatographic assay, MXene

## Abstract

Herein, a catalytic amplification enhanced dual-signal immunochromatographic assay (ICA) based on Pt nanoparticles (Pt NPs) modified with Ti_3_C_2_Tx MXene (Ti_3_C_2_Tx@Pt) was first developed for chloramphenicol (CAP) in animal-derived foods. Due to the large specific surface area and abundant active sites of Ti_3_C_2_Tx@Pt, they can be loaded with hundreds of Pt NPs to enhance their catalytic activity, resulting in a significant increase in the detection sensitivity; the sensitivity was up to 50-fold more sensitive than the reported ICA for CAP. The LODs of the developed method for milk/chicken/fish were 0.01 μg/kg, the LOQs were 0.03 μg/kg and the recovery rates were 80.5–117.0%, 87.2–118.1% and 92.7–117.9%, with corresponding variations ranging from 3.1 to 9.6%, 6.0 to 12.7% and 6.0 to 13.6%, respectively. The linear range was 0.0125–1.0 μg/kg. The results of the LC-MS/MS confirmation test on 30 real samples had a good correlation with that of our established method (R^2^ > 0.98), indicating the practical reliability of the established method. The above results indicated that an ICA based on the Ti_3_C_2_Tx@Pt nanozyme has excellent potential as a food safety detection tool.

## 1. Introduction

Chloramphenicol (CAP) is a highly effective bacteriostatic broad-spectrum antibiotic that has been widely used in the production, processing and manufacturing of livestock, poultry and aquatic products [1]. CAP residues in food can cause great harm to human health, including the following: aplastic anemia, bone marrow transplantation, leukemia, gray baby syndrome, etc. [2]. At present, the United States, the European Union and China have issued relevant laws and regulations prohibiting the use of CAP in the treatment of animal diseases, and it must not be detected in food [3]. However, due to its low cost and good effect, the illegal use of chloramphenicol is still banned repeatedly [4]. Therefore, establishing a fast and convenient method for detecting CAP residues in food plays an important role in ensuring food safety.

Currently, the detection methods of CAP in food are mainly instrumental methods, such as LC-MS/MS [5,6], GC-ECD [7,8], etc. While the above instrumental approaches are highly selective and specific, they need costly equipment and skilled operators, and the sample pretreatment process is tedious and not suitable for the quick on-site screening of large-scale samples [9,10]. Immunochromatography (ICA) based on the specific binding of an antigen (Ag) and antibody (Ab) is widely used in on-site rapid detection due to its simple operation, fast reaction speed and instant results [9]. The current signal labels used for detecting chloramphenicol (CAP) in ICA are mainly gold nanoparticles (GNPs). However, the low molar extinction coefficient and narrow particle size range limit its detection sensitivity [11]. In order to improve sensitivity, many new nanomaterials have been used to replace GNPs, including quantum dots [12], fluorescent microspheres [13], organic dyes such as crystal violet [14], etc. The above labeling materials are all single-signal outputs, which are increasingly unable to meet the demand for the trace detection of CAP in food. Nanozymes, nanomaterials with enzyme-like characteristics, are excellent signal-reporting molecules that can catalyze the conversion of chromogenic substrates into other visible colors, achieving dual-signal output. Compared with natural enzymes, nanozymes have lower preparation costs, greater stability and higher catalytic efficiency, which makes them ideal carriers for probes in immunoassays [15,16].

In recent years, MXene-based composites as nanozymes have attracted attention [17]. The excellent hydrophilicity, large specific surface area and abundant surface groups of Ti_3_C_2_Tx MXene can provide more attachment sites for the anchoring of noble metal particles and improve the catalytic performance of composites [18]. At present, MXene-based nanozyme composite materials are mainly used in fields such as biosensing [19] and catalysis [20]. For example, when MXene@Pt is under NIR-II laser irradiation, hot electrons can be excited, promoting nano-enzymatic activity. Compared to original nanozymes, MXene-based nanozymes improved tumor suppression [21]. The high number of oxygenated groups on the MXene surface can increase the loading capacity of Pt NPs, and the introduction of MXene enhances the catalytic ability of Pt NPs for Hg^2+^, enhancing their adsorption capacity for Hg^2+^ [22]. The above research results indicated that Ti_3_C_2_Tx MXene was an excellent nanomaterial loading matrix, and the addition of MXene enhanced the catalytic activity of single-atom metal particles. As a signal reporter molecule, it had broad applications in providing stable signal output and improving the stability of biosensors. At present, the application of ICA based on Pt NPs modified with MXene Ti_3_C_2_Tx is relatively limited, with only medical reports on its use for detecting HIV-DNA, and there is still a gap in small molecule detection [23].

Here, we synthesized Ti_3_C_2_Tx@Pt nanocomposites through simple in situ reduction and applied them for the detection of CAP in animal-derived foods. Due to the large specific surface area and abundant active sites of Ti_3_C_2_Tx@Pt, they can be loaded with hundreds of Pt NPs to enhance their catalytic activity, resulting in a significant increase in the detection sensitivity of ICA. The successful establishment of this method has expanded the application of MXene materials in the field of POCT.

## 2. Materials and Methods

### 2.1. Reagents and Instruments

LiF (98%), Ti_3_AlC_2_ (98%), H_2_PtCl_6_·6H_2_O (Pt ≥ 37.5%) and 3,3′,5,5′-tetramethylbenzidine (TMB) were purchased from Macklin Inc. (Macklin, Shanghai, China). Bovine serum albumin (BSA, 98%), sodium borohydride (NaBH_4_) and 3-amino-9-ethylcarbazole (AEC) were bought from Sigma-Aldrich (St. Louis, MO, USA). The polyclonal Ab against CAP (anti-CAP Ab, 50 mg/mL), goat anti-rabbit IgG (17.5 mg/mL) and coating Ag (CAP-BSA, 15 mg/mL) were prepared in our lab. The sample pads (SB08, GF-2), absorbent pads (CH37K) and polyvinyl chloride (PVC) gasket (SMA31-40) were bought from Shanghai Liangxin Co., Ltd. (Shanghai, China). The nitrocellulose membrane (UniSart CN95) was obtained from Sartorius Stedim Biotech GmbH (Goettingen, Germany).

The BioDot-XYZ3060 dispensing platform was made by BioDot Corporation (Irvine, CA, USA). The high-speed refrigerated centrifuge (GL-23M) was purchased from Hunan Xiangyi Technology Co., Ltd. (Changsha, China). The CTS300 automatic slitting machine and programmable ZQ-2000 strip cutting machine were manufactured by Shanghai Jinbiao Biotechnology, Inc. (Shanghai, China).

### 2.2. Preparation of Ti_3_C_2_Tx

The multilayer Ti_3_C_2_Tx two-dimensional nanosheets were prepared by LiF/HCl in situ etching. First, LiF (3.2 g) was dissolved in 40 mL of HCl (9 M) and stirred evenly. The as-prepared multilayer nanosheets were centrifuged (1300× *g*, 5 min) several times with ultrapure water until the supernatant reached pH 6. The precipitates containing numerous layers of nanosheets were dissolved in ultrapure water and sonicated for 2 h to prepare a thin layer of Ti_3_C_2_Tx. Finally, the supernatant colloidal dispersion containing several layers of Ti_3_C_2_Tx was collected by centrifugation (21,000× *g*, 10 min) and stored under nitrogen gas at 4 °C.

### 2.3. Preparation of Ti_3_C_2_Tx@Pt Nanocomposites

The composite material, Ti_3_C_2_Tx@Pt, was prepared using an in situ reduction strategy. Briefly, 500 μL of H_2_PtCl_6_·6H_2_O (19.3 mM) was added to 10 mL of a thin layer of the Ti_3_C_2_Tx solution and stirred vigorously for 30 min. Subsequently, 200 μL of NaBH_4_ (0.1 M) was quickly added to the reaction mixture and stirred gently for 3 h. Ti_3_C_2_Tx@Pt was then separated through centrifugation (21,000× *g*, 10 min) and dried overnight.

### 2.4. Preparation of Ti_3_C_2_Tx@Pt-Abs Probes

**Scheme 1:**
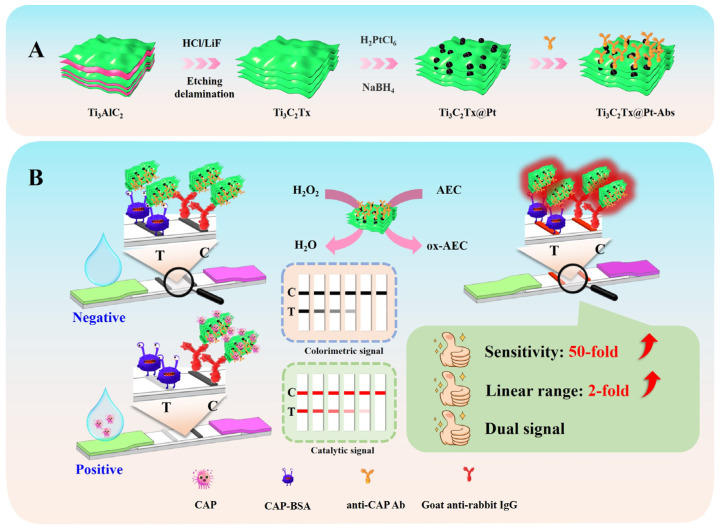
Schematic diagram of Ti_3_C_2_Tx@Pt-ICA. (**A**) Synthesis of Ti_3_C_2_Tx@Pt and Ti_3_C_2_Tx@Pt-Abs, (**B**) detection principle of Ti_3_C_2_Tx@Pt-ICA.

First, Ti_3_C_2_Tx@Pt (1 mg) was ultrasonically dispersed in phosphate buffer (PB). Subsequently, 40 μg of anti-CAP Abs was mixed into the solution and stirred lightly for 30 min. Then, 40 μL of 10% BSA was used to block the reaction for 30 min. Finally, the precipitate was redissolved in 400 μL PB (0.02 M, pH 7.4, 0.3% PVP, 5% sucrose, 0.5% BSA, 0.5% Tween^®^-20) after centrifugation for 10 min (21,000× *g*, 4 °C), which was the probe solution (Scheme 1A).

### 2.5. The Peroxidase-like Activity of Ti_3_C_2_Tx@Pt

Steady-state kinetic tests were performed by monitoring changes in the absorbance of TMB oxidation products under 652 nm. The TMB or H_2_O_2_ substrate concentration was constant, while the other substrate concentrations were varied. The concentration ranges of TMB and H_2_O_2_ were 2–28 mM and 0.2–1.6 M, respectively. The two substrates were mixed and dissolved at different concentrations in citrate disodium phosphate buffer (pH 4.5). The kinetic parameters were calculated using the Lineweaver–Burk plots of the double reciprocal of the Michaelis–Menten equation: 1/*ν* = (K_m_/*V*_max_) (1/[S] + 1/K_m_), where *ν* is the initial velocity, and *V*_max_ is the maximum reaction velocity.

### 2.6. The Preparation of the Test Strip

The test strip consists of four components: a nitrocellulose membrane (NC), PVC gasket, absorbent pads and sample pads. The sample pads were immersed in 0.1 M Borax borate buffer (BB, pH 8.0, containing 0.15 M NaCl, 0.3% Polyvinylpyrrolidone (PVP), 0.5% Tween^®^-20) and dried at 37 °C overnight. The dried sample pads and absorbent pads were cropped to 1.5 cm × 30 cm and 2.5 cm × 30 cm, respectively. The goat anti-rabbit IgG (0.12 mg/mL) and coating Ag (0.19 mg/mL) were squirted on the NC membrane to form C-line and T-line, respectively. The above prepared materials were assembled as shown in Scheme 1B and cut into 3.05 mm wide test strips.

### 2.7. Sample Pretreatment and Detection Process

**Chicken and fish:** First, the CAP-free chicken and fish samples were homogenized, and 4 g of the uniform sample was precisely weighed into a 15 mL tube. A total of 2 mL of ultrapure water and 4 mL of ethyl acetate were added and stirred vigorously for 3 min; after 10 min of centrifugation at 6800× *g*, the upper ethyl acetate layer was moved to another 10 mL tube and dried in the air at 50 °C. The mixture was then redissolved in PB buffer for testing.

**Milk:** the milk samples were tested directly without pretreatment.

**Detection process:** The above sample solution (150 μL) was added to the microwells previously coated with 4.5 μL of Ti_3_C_2_Tx@Pt-Abs probe and incubated 3 min. Placing the test paper upright in the microtiter wells, the reaction took 8 min. The catalytic reaction was then carried out by immersing the test paper in AEC substrate solution (13.3 mM) for 30 s. The color intensity of the test paper was taken with a smartphone, and the data were processed with Image J 1.53K software.

### 2.8. The Performance Assessment of the Developed Ti_3_C_2_Tx@Pt-ICA

#### 2.8.1. Sensitivity

Different concentrations of CAP were added to the samples, and each sample was tested three times to obtain the visual limit of detection (vLOD), limit of detection (LOD), the limit of quantitation (LOQ) and linear detection range. The vLOD was the lowest CAP concentration at which the T-line color intensity was weaker than the C-line color intensity. The Ti_3_C_2_Tx@Pt-ICA is based on the principle that the target and the coating Ag compete to bind limited Ab probes; the color intensity of the T-line is inversely proportional to the concentration of the target (Scheme 1B). The calibration curves were exported by plotting the logarithm of CAP concentration as the *X*-axis and B/B_0_ as the *Y*-axis (B_0_ represents the T/C value of negative samples; B represents the T/C value of positive samples). The LOD and LOQ are defined as the mean of 20 blank samples plus 3 or 10 times the standard deviation (SD). The IC_20_-IC_80_ of the calibration curve is the linear detection range.

#### 2.8.2. Selectivity

Cross-reaction (CR, %) was used to evaluate the selectivity of Ti_3_C_2_Tx@Pt-ICA. CAP and four other amide alcohol drugs, including chloramphenicol sodium succinate (CAPSS), thiamphenicol (TAP), florfenicol (FF) and florfenicol amine (FFA) were tested using the developed Ti_3_C_2_Tx@Pt-ICA and indirect competitive enzyme-linked immunosorbent assay (icELISA). The CR rates were derived from the following formula: CR (%) = IC_50 (CAP)_/IC_50 (other amide alcohol drugs)_ × 100.

#### 2.8.3. Accuracy and Precision

Three different concentrations of CAP (0.03/0.05/0.26 μg/kg, 0.03/0.05/0.27 μg/kg, 0.02/0.04/0.20 μg/kg) were spiked in milk, chicken and fish samples. All samples were collected for testing in triplicate within three days. The recovery and coefficient of variation (CV) determined the accuracy and precision of Ti_3_C_2_Tx@Pt-ICA.

#### 2.8.4. Confirmatory Test 

A total of 30 samples of chicken, fish and milk were bought at a local market (Guangzhou, China), and the quantity of each sample was confirmed to be 10 without CAP. Different levels of CAP were added to all samples; only the operator knew their actual concentration. Each sample was simultaneously tested in triplicate by Ti_3_C_2_Tx@Pt-ICA and LC-MS/MS. A detailed description can be found in the Supplementary Material for LC-MS/MS.

### 2.9. Statistical Analysis

Three repetitions of each experiment were performed (n = 3). Origin 2021 was used for statistical analysis. The significance level was defined as p less than 0.01 (*p* < 0.01). The data are shown as the average ± standard deviation (SD).

## 3. Results and Discussion

### 3.1. Characterization of Ti_3_C_2_Tx@Pt

**Figure 1:**
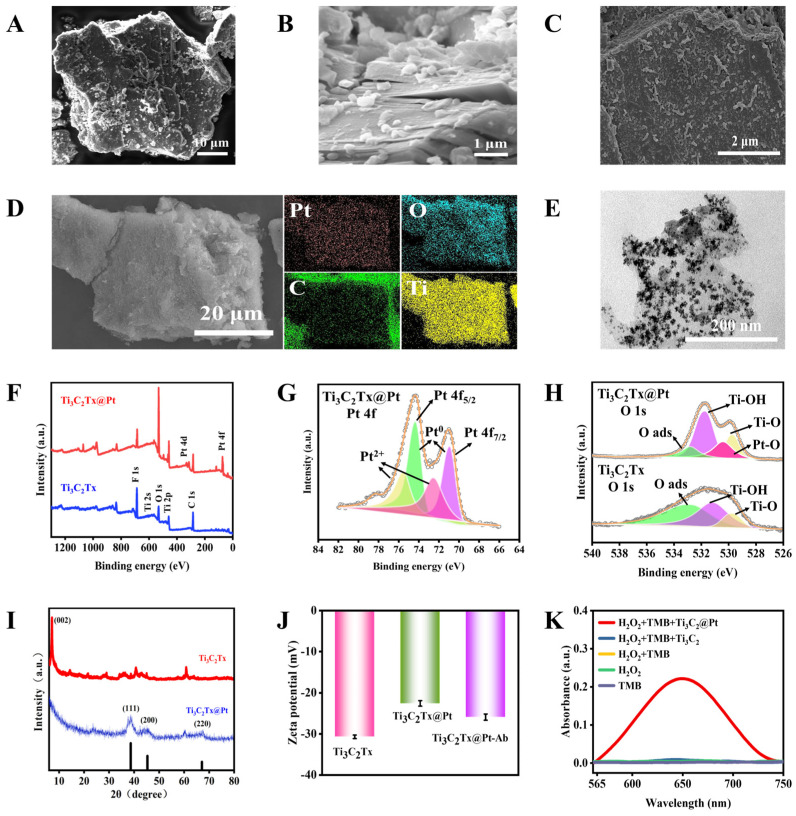
Characterization of Ti_3_C_2_Tx@Pt. SEM image of (**A**) Ti_3_AlC_2_ MXene, (**B**) Ti_3_C_2_Tx and (**C**) Ti_3_C_2_Tx @Pt nanocomposites. (**D**) EDS spectra of Ti_3_C_2_Tx @Pt nanocomposites. (**E**) TEM image of Ti_3_C_2_Tx @Pt. High-resolution XPS spectra of (**F**) Ti_3_C_2_Tx @Pt and Ti_3_C_2_Tx, (**G**) Pt 4 f and (**H**) O 1s. (**I**) XRD spectra of Ti_3_C_2_Tx and Ti_3_C_2_Tx@Pt nanocomposites. (**J**) Zeta potentials of Ti_3_C_2_Tx, Ti_3_C_2_Tx @Pt and Ti_3_C_2_Tx @Pt-Abs. (**K**) UV-vis absorption spectra of various solutions.

The dense layered structure of Ti_3_AlC_2_ was shown by scanning electron microscopy (SEM) (Figure 1A), and the sparse layered structure of Ti_3_C_2_Tx after etching (Figure 1B) was also observed, whose large surface area can load more Pt NPs. SEM (Figure 1C) and transmission electron microscopy (TEM, Figure 1E) showed that Pt NPs were highly dispersed among the layer and on the surface of Ti_3_C_2_Tx. X-ray energy spectroscopy (EDS) (Figure 1D) showed that the Ti_3_C_2_Tx@Pt nanocomposites contained Ti, O, C and Pt elements, all of which were evenly spread on the Ti_3_C_2_Tx surface, further confirming the large amount of Pt NPs loaded on Ti_3_C_2_Tx.

X-ray photoelectron spectroscopy (XPS) spectra of Ti_3_C_2_Tx and Ti_3_C_2_Tx@Pt showed the presence of elements such as Pt, C, F and O (Figure 1F). Two new peaks of Ti_3_C_2_Tx@Pt appeared in the XPS spectrum, Pt 4d and Pt 4f. As shown in the high-resolution Pt 4f spectrum of Figure 1G, two distinct peaks at the −74.3 eV and −70.9 eV binding energies correspond to 4f_5/2_ and 4f_7/2_. The peaks can be divided further into two groups, corresponding to Pt^2+^ and Pt^0^. These showed that a robust relationship exists within Ti_3_C_2_Tx with Pt NPs, which positively affects the adsorption properties of Pt NPs on the Ti_3_C_2_Tx surface. Moreover, by comparing the XPS spectra of Ti_3_C_2_Tx and Ti_3_C_2_Tx@Pt, it was found that there were three clear peaks in the O 1s spectrum of Ti_3_C_2_Tx in Figure 1H, including Ti-O and Ti-OH bonds, as well as the adsorbed oxygen in the surface sample (O ads); the binding energies were approximately 529.75 eV, 531.2 eV and 532.8 eV. Meanwhile, Ti_3_C_2_Tx@Pt can also detect the Pt-O peak with a binding energy of approximately 530.4 eV. These results further confirmed that Pt NPs were successfully loaded onto Ti_3_C_2_Tx and distributed uniformly. The X-ray diffraction results (XRD, Figure 1I) showed a peak of the highest Ti_3_AlC_2_ (Figure S1) with JCPDS card no. 52-0875 as an indicator that completely disappears at about 39.5°, and the diffraction peak of the Ti_3_C_2_Tx reflection surface (002) was observed at about 6.2°, indicating that the Al layer in Ti_3_AlC_2_ was basically completely etched. At 39.76°, 46.24° and 67.45°, the peaks of diffraction belong to the (111), (200) and (220) crystal faces of the Pt NPs, respectively (JCPDS certificate no. 040802). In addition, as shown in Figure 1J, the potential of Ti_3_C_2_Tx loaded with negatively charged Pt NPs changed from −30.7 mV to −22.57 mV, indicating that the Ti_3_C_2_Tx@Pt was successfully prepared. In particular, after coupling Abs with Ti_3_C_2_Tx@Pt, the potential changed from −22.57 mV to −25.9 mV. These results showed that the Ti_3_C_2_Tx@Pt-Abs probe had been successfully prepared.

### 3.2. Evaluation of Ti_3_C_2_Tx@Pt Peroxidase-like Enzyme Activity

The catalytic oxidation of the chromogenic substrate TMB in the presence of H_2_O_2_ produces a blue product with a characteristic adsorption peak at 652 nm to assess the peroxidase-like activity of Ti_3_C_2_Tx@Pt. Figure 1K shows that when TMB + H_2_O_2_ + Ti_3_C_2_Tx@Pt was present simultaneously, a blue-colored product with a highly distinctive absorption peak at 652 nm can be generated, while the peaks for the other groups are barely visible. It was particularly emphasized that Ti_3_C_2_Tx itself does not exhibit peroxidase-like activity (blue line). However, due to their large specific surface area and active binding sites, the loading of Pt NPs results in their strong peroxidase-like activity.

The steady-state kinetics were used to further confirm Ti_3_C_2_Tx@Pt peroxidase-like activity (Figure S3). The lower K_m_ value showed the strength of the enzyme’s affinity for the substrate [24]. Table S1 [25,26,27,28,29,30,31,32,33,34,35,36] summarized the activity of nanozymes based on Pt. The results showed that when using TMB as the substrate, the K_m_ of Ti_3_C_2_Tx@Pt (0.17 mM) was the lowest, which means that the peroxidase activity of Ti_3_C_2_Tx@Pt was the strongest.

### 3.3. Optimizing the Synthesis of Ti_3_C_2_Tx@Pt

#### 3.3.1. Optimization of H_2_PtCl_6_·6H_2_O Amount

The amount of H_2_PtCl_6_·6H_2_O had a significant impact on the loading and signal enhancement in Pt NPs on Ti_3_C_2_Tx [37]. The T-line color intensity became stronger with an increasing amount of H_2_PtCl_6_·6H_2_O, but the inhibition ratio deteriorated, as shown in Figure S2A. The color intensity and inhibition ratio reached the desired level when the amount of H_2_PtCl_6_·6H_2_O was 25 μL. Therefore, 25 μL of H_2_PtCl_6_·6H_2_O was chosen as the optimal amount of H_2_PtCl_6_·6H_2_O.

#### 3.3.2. Optimization of Synthesis Time

As the synthesis time increased, more Pt NPs were loaded onto Ti_3_C_2_Tx. As shown in Figure S2B, the loading amount of Pt NPs on Ti_3_C_2_Tx tended to reach saturation when the reaction time was 9 h. Meanwhile, the inhibition ratio and color intensity reached the ideal state. Therefore, the optimal synthesis time was 9 h.

#### 3.3.3. The Optimization of the Reductant Amount

The more reductant was used, the smaller the particles. The smaller the amount of reductant, the larger the particles, which can increase the molar absorption coefficient and enhance color development [38]. The color intensity and inhibition ratio were optimal at 60 μL of NaBH_4_, as shown in Figure S2C, so the optimal reductant amount was 60 μL.

### 3.4. Optimization of Ti_3_C_2_Tx@Pt-ICA

To achieve the best detection performance of Ti_3_C_2_Tx@Pt-ICA, we optimized various critical technical parameters, which included the coupling pH, the amount of Ab, blocking agent, catalytic reaction time, catalytic substrate and catalytic methods. 

#### 3.4.1. Optimization of Coupling pH

**Figure 2:**
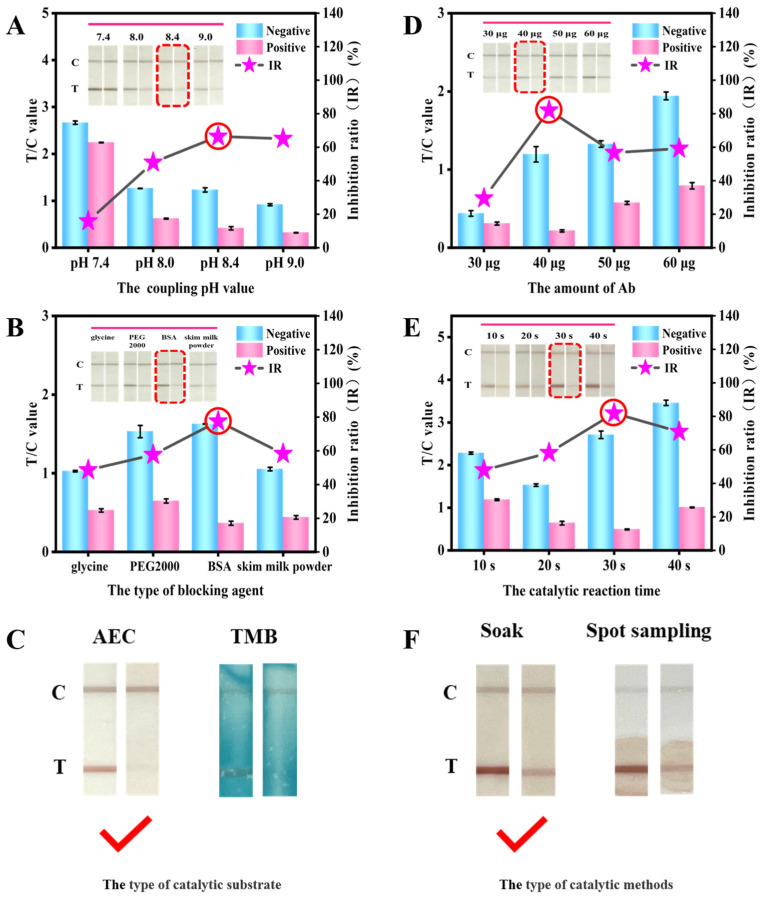
The synthesis optimization of Ti_3_C_2_Tx@Pt-ICA: including (**A**) the coupling pH value, (**B**) the amount of Ab, (**C**) the type of blocking agent, (**D**) the catalytic reaction time, (**E**) the type of catalytic substrate and (**F**) the type of catalytic methods. The inhibition ratio was calculated as indicated: inhibition ratio (%) = [(T/C_negative_ − T/C_positive_)/(T/C_negative_)] × 100.

A suitable coupling pH is beneficial for the strong binding of Ab to Ti_3_C_2_Tx@Pt, improving the ability to recognize Ag [39]. Figure 2A shows that the T-line color intensity decreased with increasing pH, but the inhibition effect became better. The desired inhibition ratio and color intensity effect were achieved at a coupling pH of 8.4. Therefore, the optimal coupling pH was 8.4.

#### 3.4.2. Optimization of Ab Amount

Too little Ab was not enough to achieve the desired color intensity on a competitive ICA. Excessive amounts of Ab will result in excess Ab binding to the coating Ag, thus reducing sensitivity [40]. Figure 2B shows the T-line color intensity increased with increasing Ab content, but the inhibition became weaker. When the Ab amount was 40 μg, the ideal color intensity and best inhibition effects were obtained. Therefore, the optimal amount of Ab was chosen to be 40 μg. 

#### 3.4.3. Optimization of Blocking Agent

A suitable blocking agent can prevent the non-specific adsorption of nanoprobes and increase the sensitivity of the approach [41]. Figure 2C shows four commonly used blocking agents (10% glycine, 10% PEG-2000, 10% BSA, 10% skim milk powder); using 10% BSA as the blocking agent resulted in a higher inhibition ratio and better color intensity. Therefore, the optimal blocking agent was chosen to be 10% BSA. 

#### 3.4.4. Optimization of Catalytic Reaction Time

The catalytic reaction time determined the effect of the signal amplification and detection sensitivity [42]. The test strip was soaked in an AEC/H_2_O_2_ solution and recorded the T-line color intensity from 10 to 40 s, respectively. As shown in Figure 2D, as the soaking time increased, the color intensity in the T-line became progressively stronger, but the inhibition effect was the best at 30 s. Thus, the optimal catalytic reaction time was chosen to be 30 s.

#### 3.4.5. Optimization of Catalytic Substrate

The amplification effect of catalytic signals varies with different catalytic substrates. The catalytic effects of TMB and AEC are shown in Figure 2E; there was a phenomenon of the dissolution and diffusion of TMB after oxidation, which was consistent with previous reports [43]. On the contrary, AEC precipitated on the T-line, producing a bright and clear red signal.

#### 3.4.6. Optimization of Catalytic Methods

The results of two different catalytic methods are shown in Figure 2F. The soaking method was more effective than the spot sampling method in enhancing the color intensity of the strips, improving the smoothness of sample running and reducing background interference. Therefore, soaking was chosen as the optimal catalytic method.

### 3.5. Performance Evaluation of Ti_3_C_2_Tx@Pt-ICA

#### 3.5.1. Sensitivity

**Figure 3:**
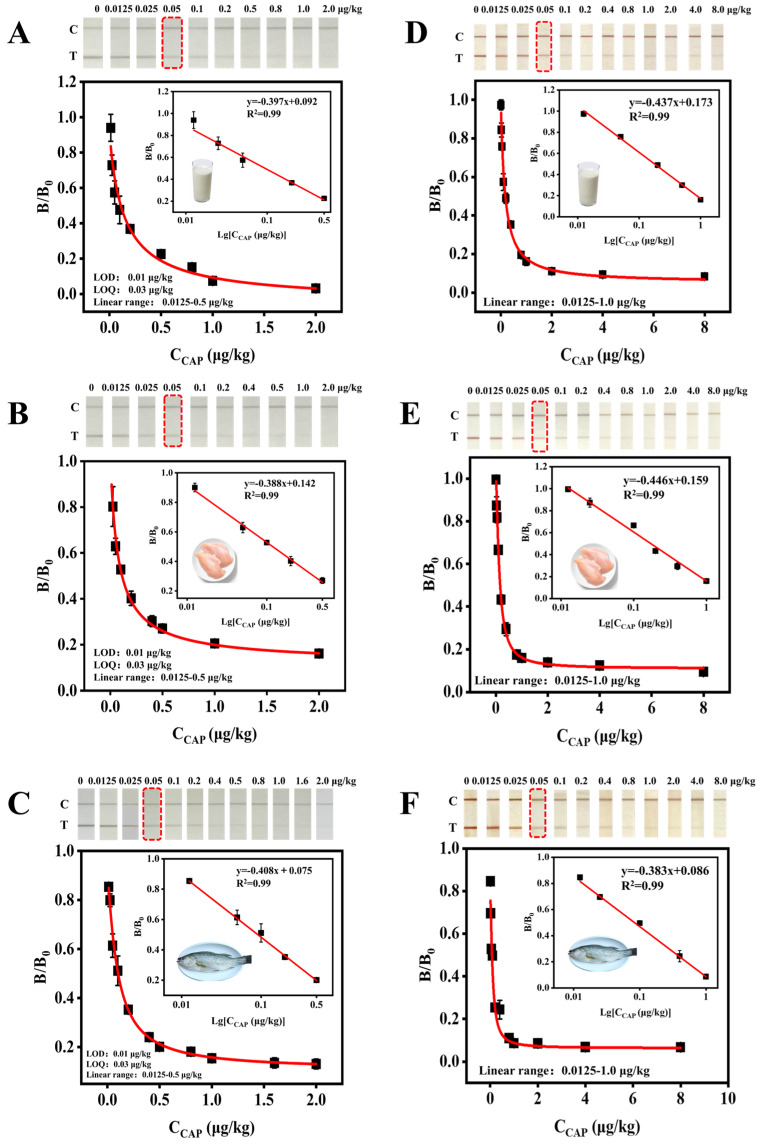
A performance evaluation of Ti_3_C_2_Tx@Pt-ICA. The calibration curves of Ti_3_C_2_Tx@Pt-ICA for the detection of CAP in (**A**) milk, (**B**) chicken, (**C**) fish, respectively. The calibration curves of catalytic signals for the detection of CAP in (**D**) milk, (**E**) chicken, (**F**) fish, respectively.

Figure 3A–C show that the vLODs of the developed method for milk, chicken and fish were 0.05 μg/kg, LODs were 0.01 μg/kg and LOQs were 0.03 μg/kg, respectively. The linear ranges were 0.0125–1.0 μg/kg (Figure 3D–F).

#### 3.5.2. Selectivity

Table S2 shows the selectivity results for Ti_3_C_2_Tx@Pt-ICA. CAP Ab showed a high CR on CAPSS which was 160.8%. This may be due to the similarity in structure between CAPSS and CAP [44]. There was almost no CR for FF, FFA and TAP, and the CR value was below 0.1%. The results indicated that Ti_3_C_2_Tx@Pt-ICA can simultaneously detect CAPSS and CAP.

#### 3.5.3. Accuracy and Precision

Table S3 shows that the recovery rates of Ti_3_C_2_Tx@Pt-ICA were 80.5–117.0%, 87.2–118.1% and 92.7–117.9%, respectively. The corresponding CVs were 3.1–9.6%, 6.0–12.7% and 6.0–13.6%, respectively. The above results confirm that the established Ti_3_C_2_Tx@Pt-ICA exhibited excellent accuracy and reproducibility.

#### 3.5.4. Confirmatory Test

Table 1 shows that the developed method was in good agreement with LC–MS/MS, with a significant correlation rate of 0.98 (Figure S4). The data showed that the method we developed had excellent reliability for rapid testing CAP in animal tissues.

### 3.6. Comparison of ICA for CAP Detection

Table 2 summarizes the ICAs used to detect CAP. Firstly, on the ICA platform, the most commonly used labeling material was GNPs. This study prepared a Ti_3_C_2_Tx@Pt nanocomposite with peroxidase-like activity as the signal amplification label. The loading of noble metal Pt NPs enhanced the enzyme activity of Ti_3_C_2_Tx@Pt, giving it significant advantages in expanding the detection range. The linear range was doubled. Secondly, our test can detect milk, chicken and fish at the same time, while most of the approaches described previously can only detect one or two of them. Thus, the range of samples monitored was substantially increased by our method. Finally, the Ti_3_C_2_Tx@Pt nanocomposite had a large specific surface area and could carry more Ab, which increased the detection sensitivity up to 50-fold compared to the reported GNPs-ICA. Therefore, this method had significant advantages in terms of the linear detection range, sample adaptability and sensitivity. However, the material we used is still in the preliminary research stage compared to the traditional GNPs, and there is still a long way to go before commercialization. Therefore, strengthening the research of this material in the field of food safety testing, leveraging its advantages in sensitivity, stability, etc. and accelerating its commercialization process are the research directions we will strive for in the future.

## 4. Conclusions

In this study, Ti_3_C_2_Tx@Pt composites with excellent peroxidase-like activity were successfully synthesized, and AEC-based Ti_3_C_2_Tx@Pt-ICA was prepared for ultrasensitive CAP detection in milk, chicken and fish. The excellent enzyme-like activity of Ti_3_C_2_Tx@Pt was attributed to the abundance of oxygenated groups on the Ti_3_C_2_Tx MXene surface and the large specific surface area, which enabled it to load hundreds of Pt NPs. Ti_3_C_2_Tx@Pt can catalyze the color development of the chromogenic substrate AEC and achieve the catalytic amplification of the ICA signal. The developed Ti_3_C_2_Tx@Pt-ICA showed LODs and LOQs of 0.01 μg/kg and 0.03 μg/kg for milk, fish and chicken, respectively. Ti_3_C_2_Tx@Pt-ICA was 50-fold more sensitive than the reported GNPs-ICA. Finally, the Ti_3_C_2_Tx@Pt-ICA sensing platform was successfully applied to real sample detection. The results of the LC-MS/MS confirmation test on 30 real samples had a good correlation with that of our established method (R^2^ > 0.98). This result tells us that in practical applications, we can first use our established method for rapid screening, and the suspected positive samples screened out can be confirmed by confirmation methods such as LC-MS/MS, which can greatly improve detection efficiency and reduce detection costs. Our research has pioneered the application of MXene (Ti_3_C_2_Tx@Pt) in the field of food safety detection, providing a new signal label for the establishment of high-performance ICA.

## Figures and Tables

**Table 1:** Determination of CAP in milk, chicken and fish by LC-MS/MS and Ti_3_C_2_Tx@Pt-ICA (n = 3).

Sample No.	LC-MS/MS (μg/kg)	CV (%)	Ti_3_C_2_Tx@Pt–ICA (μg/kg)	CV (%)
Milk1	ND	-	ND	-
Milk2	0.16	3.13	0.16	13.87
Milk3	1.34	2.24	1.36	8.06
Milk4	ND	-	ND	-
Milk5	ND	-	ND	-
Milk6	0.15	-	0.15	6.73
Milk7	0.30	1.67	0.29	9.57
Milk8	ND	-	ND	-
Milk9	0.31	-	0.32	6.86
Milk10	1.28	2.34	1.37	9.99
Chicken1	0.21	2.38	0.28	3.06
Chicken2	ND	-	ND	-
Chicken3	0.25	-	0.24	6.96
Chicken4	2.33	1.72	1.80	10.75
Chicken5	ND	-	ND	-
Chicken6	0.53	2.83	0.51	6.19
Chicken7	ND	-	ND	-
Chicken8	2.07	1.69	2.20	3.44
Chicken9	0.46	3.26	0.49	8.82
Chicken10	ND	-	ND	-
Fish1	2.13	1.41	2.58	10.48
Fish2	0.25	2.00	0.23	1.17
Fish3	ND	-	ND	-
Fish4	0.25	-	0.25	5.80
Fish5	2.12	2.59	2.85	3.31
Fish6	ND	-	0.016	5.95
Fish7	0.51	0.98	0.56	9.73
Fish8	ND	-	ND	-
Fish9	0.54	1.85	0.57	2.59
Fish10	ND	-	ND	-

ND: not detected; -: unavailable.

**Table 2:** Comparison of ICAs for detecting CAP in animal-derived foods.

Methods	Sample	vLOD (μg/kg)	LOD (μg/kg)	Reference
DANS-ICA	Milk/Honey	3	-	[1]
MGNPs-ICA	Fish	-	0.235	[45]
NAN-ICA	Milk	3	0.098	[46]
CV-ICA	Milk/Honey	2	-	[14]
LFM-ICA	Honey/Egg/Fish	0.25	0.048/0.061/0.073	[13]
QDs-ICA	Milk	0.1	0.2	[47]
QDs-ICA	Milk	-	0.12	[48]
QDs-ICA	Chicken	-	0.016	[12]
GNPs-ICA	Honey	5	0.02	[49]
GNPs-ICA	Milk	0.5	-	[44]
GNPs-ICA	Milk	5	0.5	[50]
GNPs-ICA	Milk	1.2	0.04	[51]
GNPs-ICA	Milk	2.4	0.016	[52]
Ti_3_C_2_Tx@Pt-ICA	Milk/Chicken/Fish	0.05	0.01	**This work**

-: unavailable or undetectable; DANS: an NR-based instant immune network label strategy; MGNPs: a multiplex immunochromatographic strip biosensor based on gold nanoparticles; NAN: a natural antibody network; LFMs: lanthanide fluorescent microspheres; QDs: quantum dots; GNPs: gold nanoparticles; CV: crystal violet.

## Data Availability

The original contributions presented in the study are included in the article/Supplementary Material, further inquiries can be directed to the corresponding authors.

## Supplementary Materials

The following supporting information can be downloaded at https://www.mdpi.com/article/10.3390/foods13091416/s1, Figure S1: XRD spectra of Ti_3_AlC_2_. Figure S2: The optimization of Ti_3_C_2_Tx@Pt synthesis: (A) the amount of H_2_PtCl_6_ 6H_2_O, (B) the synthesis time of Ti_3_C_2_Tx@Pt and (C) the amount of reductant. Figure S3: A steady-state kinetic assay of Ti_3_C_2_Tx@Pt: (A) the double reciprocal plots between reaction velocity and TMB concentration and (B) the TMB concentration dependence of the initial reaction velocity (*v*). Figure S4: Correlation analysis between Ti_3_C_2_Tx@Pt-ICA and LC-MS/MS. Table S1: Comparison of the K_m_ and *V*_max_ of different enzymes; Table S2: The IC_50_ and CR values of icELISA and Ti_3_C_2_Tx@Pt-ICA (n = 3); Table S3: The recovery of Ti_3_C_2_Tx@Pt-ICA for the detection of CAP in milk, chicken and fish samples (n = 3).
