# Supplementary material for: Ultrasensitive Ti3C2Tx@Pt-Based Immunochromatography with Catalytic Amplification and a Dual Signal for the Detection of Chloramphenicol in Animal-Derived Foods
Source: Foods. 2024 May 5;13(9):1416. doi: 10.3390/foods13091416 (PMC11083481; doi:10.3390/foods13091416)
Supplement: Supplementary file 1 [file foods-13-01416-s001.zip › foods-2986317-supplementary.pdf]

Supplementary Material

Ultrasensitive Ti3C2Tx@Pt-Based Immunochromatography with Catalytic Amplification and a Dual Signal for the Detection of Chloramphenicol in Animal-Derived Foods

Mengfang Lin [1,†], Zhimin Gao [2,†], Zhenjie Qian [3], Youwen Deng [1], Yanhong Chen [3], Yu Wang [3,*] and Xiangmei Li [1,*]

[1] Guangdong Provincial Key Laboratory of Food Quality and Safety, College of Food Science, South China Ag-ricultural University, Guangzhou 510642, China; 15978008477@163.com (M.L.); d635921341@163.com (Y.D.)
[2] Guangdong Agricultural Product Quality and Safety Center (Guangdong Green Food Development Center), Guangzhou 510230, China; 18922147530@163.com
[3] Guangzhou Institute for Food Inspection, Guangzhou 511410, China; qianzhenjie@hotmail.com (Z.Q.); chenyanhom@outlook.com (Y.C.)
* Correspondence: xxwangyu@163.com (Y.W.); lixiangmei12@163.com (X.L.); Tel.: +86-20-8528-3925 (Y.W. & X.L.)
† These authors contributed equally to this work.

LC-MS/MS analysis

The sample preparation method of LC-MS/MS was as follows: the samples (2 g) were extracted with 10 mL of ethyl acetate (containing 0.6 mL of ammonia and 5 mL of 4% NaCl) for 10 min. The supernatants were centrifuged at 6800×g for 3 min. The supernatant was transferred to a 15 mL centrifuge tube for nitrogen blowing. After drying, 2 mL of ultrapure water and 3 mL of n-hexane were added and thoroughly mixed, and then centrifuged at 6800×g for 3 min to remove fat from the sample. Subsequently, the supernatant was purified through a syringe filter (0.22 μm).

LC-MS/MS analysis was performed in multiple reaction monitoring (MRM) mode on a Shimadzu (Nexera x2, Japan) LC system coupled with an AB Sciex triple quadrupole EMR (QTRAP®4500, USA). Chromatographic separation was performed on an Agilent C18 column (InfinityLab Poroshell 120 EC-C18, 100 mm*3 mm 2.6-Micron), and the column temperature was maintained at 40°C. The mobile phase was constituted of H_2O (mobile phase A) and 100% acetonitrile (mobile phase B). The gradient elution procedure was as follows: 0-1 min, 20% B; 1-3 min, 20%-50% B; 3-6 min, 50%-65% B; 7-7.1 min, 65%-20% B. The mobile phase flow rate was 300 μL/min; the injection volume of the sample was 2 μL. The condition of mass spectrometer was as follows: ion mode, negative ionization; ion source, electrospray ionization (ESI); temperature, 500°C; curtain gas, 35 psi; ion source gas 1, 55 psi; ion source gas 2, 55 psi;

collision gas (CAD), medium; ion spray voltage, -4500 V. MS acquisition was performed using multiple reaction monitoring (MRM) mode, and the MRM parameters included MRM transition 321.1/152.1* and 321.1/257.1 (m/z, *Quantitative ion pair), declustering potential both -70 V, and collision energy -24.1 and -16.1 eV, respectively.

Figure captions

Figure S1 XRD spectra of Ti_3AlC_2.

Figure S2 The optimization of Ti_3C_2Tx@Pt synthesis: (A) the amount of H_2PtCl_6 $6H_2O$, (B) the synthesis time of Ti_3C_2Tx@Pt, (C) the amount of reductant.

Figure S3 Steady-state kinetic assay of Ti_3C_2Tx@Pt: (A) the double reciprocal plots between reaction velocity and TMB concentration, (B) the TMB concentration dependence of initial reaction velocity (*v*).

Figure S4 Correlation analysis between Ti_3C_2Tx@Pt-ICA and LC-MS/MS.

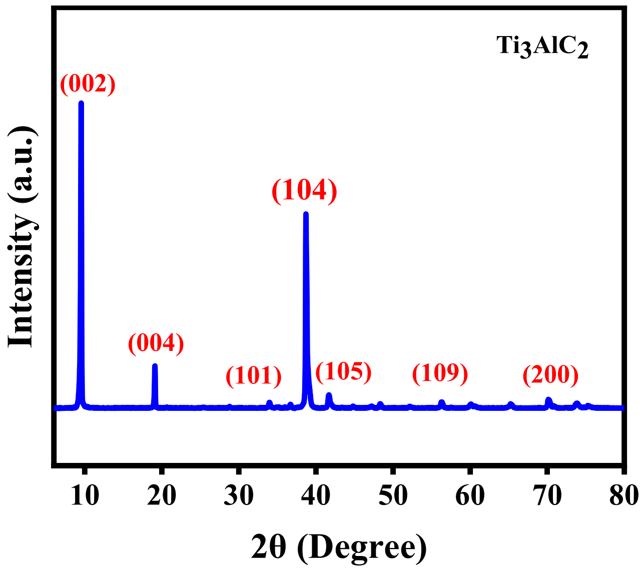

Ti3AlC2
(002)
(104)
(004)
(101)
(105)
(109)
(200)
Intensity (a.u.)
10
20
30
40
50
60
70
80
2θ (Degree)

Figure S1

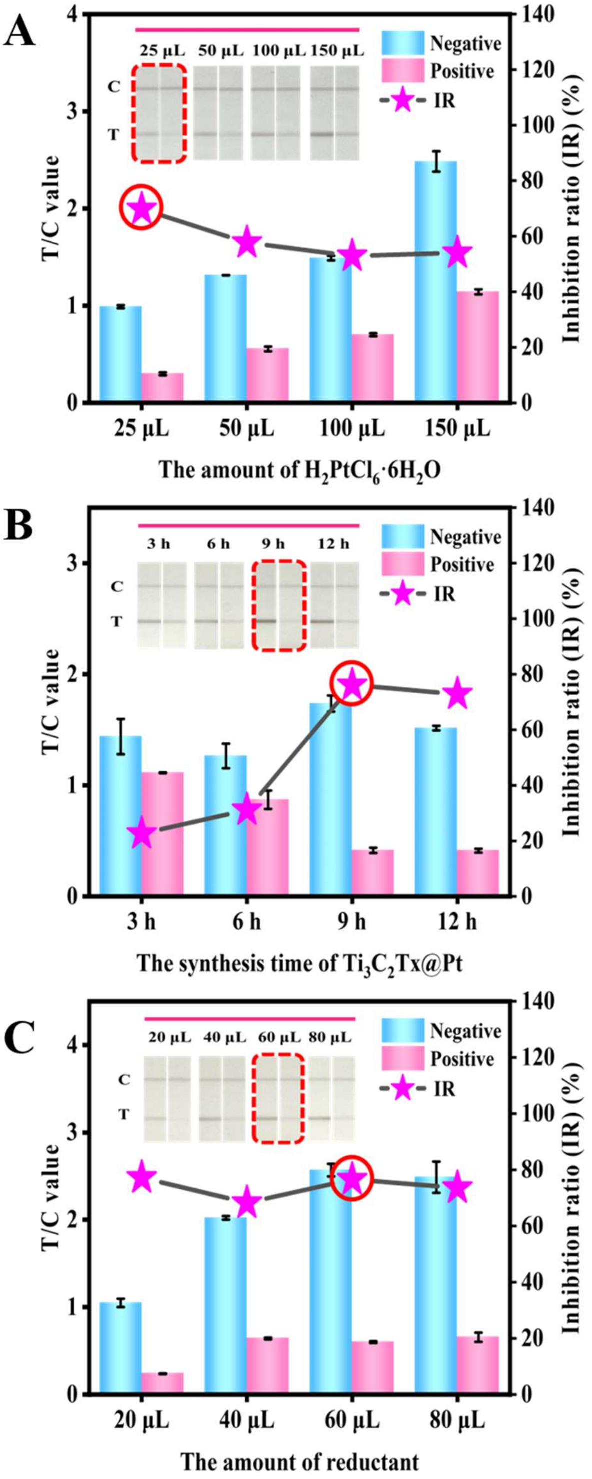
A
B
C
Negative
Positive
IR
T/C value
Inhibition ratio (IR) (%)
25 μL
50 μL
100 μL
150 μL
The amount of $H_2PtCl_6 \cdot 6H_2O$
3 h
6 h
9 h
12 h
The synthesis time of $Ti_3C_2Tx@Pt$
20 μL
40 μL
60 μL
80 μL
The amount of reductant

Figure S2

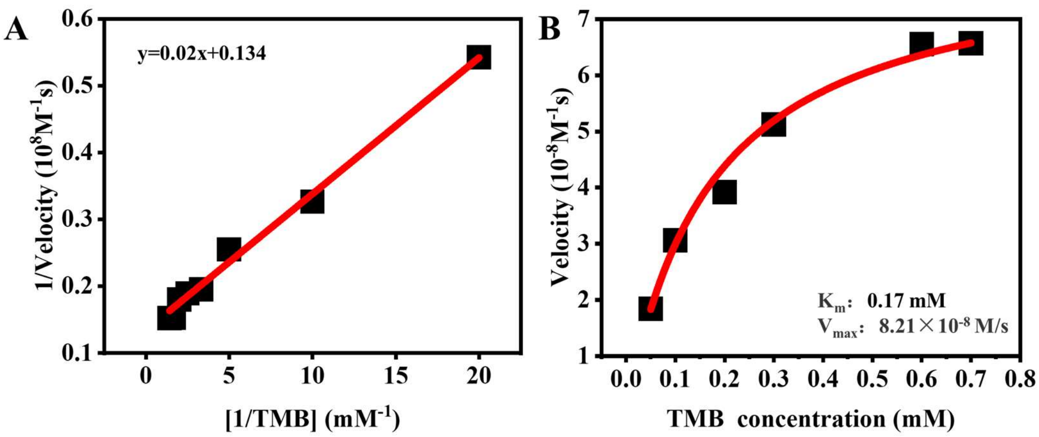

A
y=0.02x+0.134
1/Velocity (10^8M^-1s)
[1/TMB] (mM^-1)
B
Velocity (10^-8M^-1s)
Km: 0.17 mM
Vmax: 8.21×10^-8 M/s
TMB concentration (mM)

Figure S3

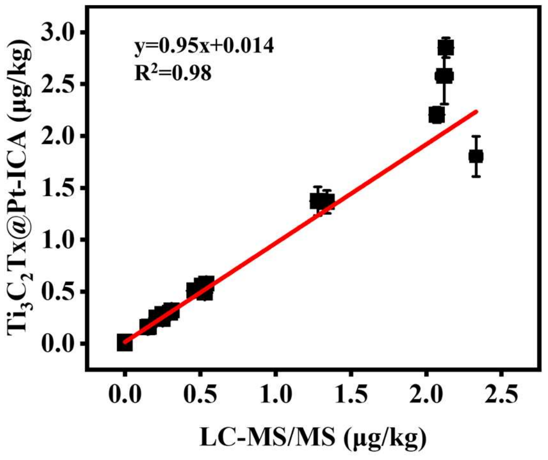

y=0.95x+0.014
R^2=0.98
Ti_3C_2Tx@Pt-ICA (μg/kg)
LC-MS/MS (μg/kg)
0.0
0.5
1.0
1.5
2.0
2.5
3.0

Figure S4

Table S1 Comparison of the K_m and V_{max} of different enzymes

Enzyme or enzyme mimic	K_m (mM)	V_{max} ($10^{-8} Ms^{-1}$)	Reference
	TMB		
HRP	0.43	10.00	[25]
Pt-Ir	7.01	10.7	[26]
PtNPs/GO	0.19	10.2	[27]
Ptn-JP NCs	0.719	51.33	[28]
Pt/PCN	1.06	23.12	[29]
Ag-Pt/rGO	3.24	20	[30]
Au@Pt	2.431	4.425	[31]
Pt NPs	0.12	126	[32]
Pd@Pt	0.516	72.1	[33]
Ps-Pt	0.3742	-	[34]
BP-Pt	0.27	13.72	[35]
FNA-Ag@Pt	1.8	7030	[36]
Ti_3C_2Tx@Pt	0.17	8.21	**This work**

-: unavailable or undetectable.

Table S2 The IC_{50} and CR values of icELISA and Ti_3C_2Tx@Pt-ICA (n=3)

**Analytes**	**Structural formula**	**icELISA**		**Ti_3C_2Tx@Pt-ICA**	
		**IC_{50} (μg/kg)**	**CR (%)**	**IC_{50} (μg/kg)**	**CR (%)**
CAP	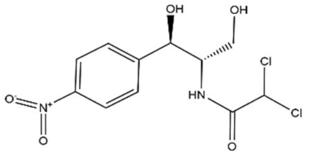	0.089	100.0	0.074	100.0
CAPSS	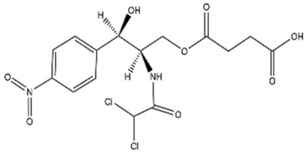	0.060	148.3	0.046	160.8
TAP	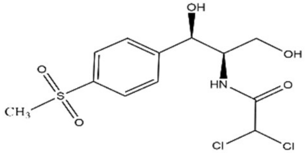	＞1000	＜0.01	> 1000	< 0.01
FF	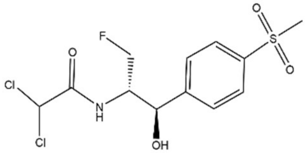	＞1000	＜0.01	> 1000	< 0.01
FFA	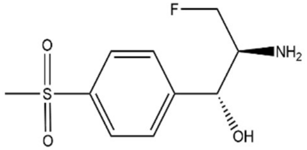	＞1000	＜0.01	> 1000	< 0.01

Table S3 Recovery of the Ti_3C_2Tx@Pt-ICA for the detection of CAP in milk, chicken, and fish samples (n=3)

**Sample**	**Spiked level (μg/kg) (Colorimetric signal)**	**Detected level (μg/kg)**	**Recovery (%)**	**CV (%)**	**Spiked level (μg/kg) (Catalytic signal)**	**Detected level (μg/kg)**	**Recovery (%)**	**CV (%)**
Milk	0.03	0.02±0.002	80.5	7.4	0.18	0.16±0.02	89.9	13.5
	0.05	0.04±0.001	90.0	3.1	0.36	0.29±0.01	82.9	4.1
	0.26	0.30±0.095	117.0	9.6	1.80	1.90±0.19	104.4	10.5
Chicken	0.03	0.02±0.003	87.2	12.7	0.16	0.13±0.01	83.7	3.8
	0.05	0.06±0.006	109.6	10.6	0.32	0.27±0.02	85.9	7.1
	0.27	0.32±0.019	118.1	6.0	1.60	1.56±0.24	97.9	15.7
Fish	0.02	0.02±0.002	92.7	10.9	0.15	0.12±0.01	82.3	4.9
	0.04	0.05±0.002	117.9	6.0	0.30	0.25±0.09	85.8	9.2
	0.20	0.21±0.029	109.1	13.6	1.50	1.29±0.17	86.1	12.9

References

25. Gao, L.; Zhuang, J.; Nie, L.; Zhang, J.; Zhang, Y.; Gu, N. Intrinsic peroxidase-like activity of ferromagnetic nanoparticles. *Nat. Nanotechnol.* **2007**, *2*, 577-83. https://doi.org/10.1038/nnano.2007.260.
26. Yang, H.; He, Q.; Pan, J.; Shen, D.; Xiao, H.; Cui, X. A Pt–Ir nanocube amplified lateral flow immunoassay for dehydroepiandrosterone. *Analyst* **2021**, *146*, 2726-33. https://doi.org/10.1039/D0AN02293D.
27. Zhang, L.; Deng, H.; Lin, F.; Xu, X.; Weng, S. A. Liu. In situ growth of porous platinum nanoparticles on graphene oxide for colorimetric detection of cancer cells. *Anal. Chem.* **2014**, *86*, 2711-8. https://doi.org/10.1021/ac404104j.
28. Guo, X.; Suo, Y.; Zhang, X.; Cui, Y.; Chen, S.; Sun, H. Ultra-small biocompatible jujube polysaccharide stabilized platinum nanoclusters for glucose detection. *Analyst* **2019**, *144*, 5179-85. https://doi.org/10.1039/C9AN01053J.
29. Shi, W.; Fan, H.; Ai, S.; Zhu, L. Honeycomb-like nitrogen-doped porous carbon supporting Pt nanoparticles as enzyme mimic for colorimetric detection of cholesterol. *Sens. Actuators, B* **2015**, *221*, 1515-22. https://doi.org/10.1016/j.snb.2015.06.157.
30. Kong, F.; Li, R.; Zhang, S.; Wang, Z.; Li, H.; Fang, H. Nitrogen and sulfur co-doped reduced graphene oxide-gold nanoparticle composites for electrochemical sensing of rutin. *Microchem. J.* **2021**, *160*, 105684. https://doi.org/10.1016/j.microc.2020.105684.
31. Wei, D.; Zhang, X.; Chen, B.; Zeng, K. Using bimetallic Au@Pt nanozymes as a visual tag and as an enzyme mimic in enhanced sensitive lateral-flow immunoassays: Application for the detection of streptomycin. *Anal. Chim. Acta.* **2020**, *1126*, 106-13. https://doi.org/10.1016/j.aca.2020.06.009.
32. Gao, Z.; Xu, M.; Hou, L.; Chen, G.; Tang, D. Magnetic bead-based reverse colorimetric immunoassay strategy for sensing biomolecules. *Anal. Chem. 2013*, *85*, 6945-52. https://doi.org/10.1021/ac401433p.
33. Wang, X.; Zhang, M.; Pang, X.; Huang, K.; Yao, Z.; Mei, X. Comparative study of Pd@Pt nanozyme improved colorimetric N-ELISA for the paper-output portable detection of Staphylococcus aureus. *Talanta* **2022**, *247*, 123503. https://doi.org/10.1016/j.talanta.2022.123503.
34. Hu, J.; Tang, F.; Wang, L.; Tang, M.; Jiang, Y.; Liu, C. Nanozyme sensor based-on platinum-decorated polymer nanosphere for rapid and sensitive detection of Salmonella typhimurium with the naked eye. *Sens. Actuators, B* **2021**, *346*, 130560. https://doi.org/10.1016/j.snb.2021.130560.
35. Li, S.; Wen, W.; Guo, J.; Wang, S.; Wang, J. Development of non-enzymatic and photothermal immuno-sensing assay for detecting the enrofloxacin in animal derived food by utilizing black phosphorus-platinum two-dimensional nanomaterials. *Food Chem.* **2021**, *357*, 129766. https://doi.org/10.1016/j.foodchem.2021.129766.
36. Du, Z.; Zhu, L.; Wang, P.; Lan, X.; Lin, S.; Xu, W. Coordination-driven one-step

rapid self-assembly synthesis of dual-functional Ag@Pt nanozyme. *Small* **2023**, *19*, 2301048. https://doi.org/10.1002/smll.202301048.
